# Population size estimation of transgender women and men in Bhutan

**DOI:** 10.1371/journal.pone.0271853

**Published:** 2022-10-07

**Authors:** Lekey Khandu, Kinley Kinley, Yonten Choki Norbu, Tashi Tobgay, Tashi Tsheten, Tenzin Gyeltshen, Sonam Choden, Willi McFarland

**Affiliations:** 1 Communicable Disease Division, Department of Public Health, Ministry of Health, National HIV, AIDS and STIs Control Program, Thimphu, Bhutan; 2 National HIV, AIDS, and STIs Control Program, Health Information and Service Center, Thimphu, Bhutan; 3 Institute of Health Partners, Thimphu, Bhutan; 4 Queer Voices of Bhutan, Thimphu, Bhutan; 5 Pride Bhutan, LGBTIQ Network of Bhutan, Thimphu, Bhutan; 6 San Francisco Department of Public Health, San Francisco, CA, United States of America; HIV/STI Surveillance Research Center and WHO Collaborating Center for HIV Surveillance, Institute for Future Studies in Health, Kerman University of Medical Sciences, ISLAMIC REPUBLIC OF IRAN

## Abstract

**Introduction:**

Transgender persons experience health disparities and are marginalized in many societies worldwide. Even their numbers are unknown in many countries. We conducted the first effort to estimate the population size of transgender women (TGW) and transgender men (TGM) in Bhutan from November 2019 to January 2020.

**Methods:**

Community-based surveys of TGW and TGM integrated several methods to estimate the size of hidden populations, including key informant mapping, wisdom-of-the-crowd, the service multiplier, and the unique object multiplier. Results of the several methods were synthesized using a Bayesian approach.

**Results:**

Surveys included 34 TGW and 124 TGM. TGW was persons assigned to the male sex at birth and currently self-identified as “trans women” (91%), “women” (6%), or another gender (3%). TGM were persons assigned female sex at birth and self-identified as “trans men” (100%). Bayesian synthesis of the multiple methods estimated 84 TGW (credible interval 61–110) and 166 TGM (credible interval 124–211) in Bhutan.

**Conclusions:**

Our study documented that TGW and TGM are part of Bhutanese society, with TGW constituting 0.03% of adult women and TGM 0.06% of adult men. Estimates can help advocate for resources and programs to address the health and well-being of these communities.

## Introduction

Throughout the world, transgender persons face multiple barriers to health and social welfare services due to gender-based stigma, discrimination, and competing survival needs [1–4]. These factors may impact transgender persons’ desire and ability to access appropriate care. Transgender women (TGW) have been shown to bear a disproportionate burden of HIV across all continents and a high prevalence of clinical depression, anxiety, and somatization [1–2]. To date, transgender persons remain under-represented in health research [1,5].

The HIV epidemic in Bhutan has so far been low-level [6]. However, is not known whether many infections are undiagnosed and concentrated in key populations, as it is in other countries in Asia [7]. Limited data suggest that the conditions for the spread of HIV exist among key populations identified by Bhutan’s National Strategic Plan for HIV 2017–2023 [6,8–11]. These key populations include female sex workers, people who inject drugs, men who have sex with men, and transgender persons. Qualitative investigations describe the emergence of sex work and the proliferation of transactional sex with recent societal changes in Bhutan [6]. Other studies have found multiple sex partners, low condom use in all types of partnerships, rising incidence of sexually transmitted infections (STIs), and difficulties in accessing HIV prevention and other health services due to sexual and gender-related discrimination [9–11]. A particular challenge to tracking the HIV epidemic has been that key population status is generally not recorded in HIV testing data, surveillance reports, or clinical records in Bhutan.

Gathering information on transgender populations faces additional challenges. Stigma, discrimination, and violence may be severe for transgender persons, as extensively reported worldwide [3,4], preventing them from accessing services or disclosing their transgender status to program providers. Therefore, it is not known if TGW in Bhutan experiences the disproportionate burden of HIV that persists in other countries [1]. Less is known about transgender men (TGM) in Bhutan, Asia, and globally [1,5].

As the first step to better understanding the HIV epidemic among transgender persons in Bhutan, the present study sought to fill the important data gap in the estimated number of transgender persons in the country. This basic information is required to efficiently allocate limited resources, set targets for care and prevention program activities, track progress on goals for HIV elimination, and advocate for laws and policies to address discrimination toward transgender persons.

## Material and methods

### Setting and study site selection

The Kingdom of Bhutan is a small landlocked country located between Tibet, China in the north and India in the south. In 2017, the total population over the age of 18 years was 537,728 [12]. The country is divided into 20 districts. The most populous and urbanized districts are the capital Thimphu (105,875 adults) and Pheuntsholing (Chhukha district, 51,888 adults) on the main border crossing with India. Data were collected in the major towns of nine districts of Bhutan (Thimphu, Phuentsholing, Wangdue Phodrang, Sarpang, Paro, Samdrup-Jongkhar, Mongar, Punakha, and Bumthang). These nine districts were selected because they encompass nearly two-thirds of all adult women (64%) and men (64%) and over four-fifths of urban women (82%) and men (81%) in Bhutan. These districts also include the three major regions of the country: central, southern border, and the more remote east.

### Study populations

For the population size estimation and eligibility for the community-based surveys, TGW and TGM were defined as persons who currently identify as a gender different from the one implied by their sex assigned at birth regardless of gender-affirming procedures, hormone use, public gender presentation, and sexual orientation. As noted with the first use above, in this report we use the abbreviations of TGW and TGM corresponding to the MeSH term “transgender person”. We note below that the terms most commonly used by participants to self-identify were “transwoman” and “transman” in English. Historical terms in Dzongkha, the official language of Bhutan, for transgender persons were not typically used by participants or peer staff in the course of this study.

### Data collection and analysis

The study was conducted from 13 November 2019 to 31 January 2020. Following UNAIDS guidelines and similar studies [13–16], we applied several methods to generate robust population size estimates and minimize the potential impact of bias resulting from a single method. Population size estimation methods included key informant mapping [17,18], wisdom-of-the-crowd [15,19], the service multiplier method [20], and the unique object multiplier method [14]. Results generated from these methods were synthesized using a Bayesian approach called the Anchored Multiplier [21,22]. The study protocol was reviewed and approved by the Research Ethics Board of Health of Bhutan. Participants provided verbal consent. No names or other identifying information were collected.

### Peer-directed community-based survey methods

Community-based surveys of TGW and TGM in Bhutan were conducted to implement components of the population size estimation methods. The surveys sampled transgender persons in the nine study districts using a peer-directed recruitment approach [16]. Peer outreach workers contacted eligible TGW and TGM in their networks to introduce the study and to encourage them to refer other eligible persons from their networks. In addition, peer outreach workers recruited participants from physical and online venues catering to LGBT populations in Bhutan by intercepting persons present at peak hours of attendance. The resulting sample was a hybrid of referral and venue-based recruitment methods [23]. Persons who were eligible and provided verbal informed consent underwent a structured, face-to-face interview that incorporated questions related to the population size estimation methods. Participants were given cell phone airtime cards of ngultrum 500 value (USD 6.79) for completing the survey and ngultrum 200 (USD 2.72) for each referral.

### Key informant mapping methods

Key informant interviews and focus group discussions were used to arrive at estimates of the numbers of TGW and TGM associated with venues catering to these communities. Details of the methods were previously reported for estimating the number of female sex workers in Bhutan [16] and described in detail by Emmanuel *et al* [17] and Ndayongeje *et al* [18]. In brief, primary, secondary, and stakeholder key informants were asked to identify all physical and online venues where TGW and TGM socialize. Primary key informants were transgender persons known to peer outreach workers or referred by other key informants. Secondary key informants were persons who had special knowledge of transgender populations (e.g., club or bar owners). Stakeholder key informants were persons who provide services to TGW and TGM, such as directors and counsellors of AIDS service organizations or human rights advocates. After listing all places where TGW and TGM socialize, key informants were asked to estimate the numbers affiliated with each venue, while counting each TGW and TGM only once if they frequented multiple venues. The median key informant response was used for each venue and the sum of the medians of all venues was the total population size. The sum of the low estimates and the sum of the high estimates was used as the range. Interviews and focus group discussions were not recorded; handwritten notes were taken without identifying individuals.

### Wisdom-of-the-crowd methods

The wisdom-of-the-crowd population size estimation method polls informed individuals (i.e., members of the target population) on their estimate of the number of persons in the hidden population [15,19,24,25]. To obtain the wisdom-of-the-crowd estimate, all survey participants were asked, respectively for their population: “If you had to guess, how many [trans women/trans men] do you think there are in Bhutan?” The mean of all responses was used as the population size and the 95% confidence interval (CI) as the plausible range. Of note, the mean was used because the 25^th^, 50^th^, and 75^th^ percentiles were the same number.

### Service multiplier methods

The service multiplier method uses a count of the number of clients from the population and a survey to determine what fraction this count represents of the whole population [14,15,20,25]. The population size estimate is given by the formula: total number = the number of clients receiving the specific service ÷ the proportion of survey respondents who report receiving the service. A 95% CI is calculated using the general capture-recapture method [15]. In the present study, the client count was the number of TGW and TGM registered with Pride Bhutan, an LGBT community-based organization. In the survey, all participants were asked: “Are you a member of Pride Bhutan?”

### Unique object multiplier methods

The unique object multiplier method is similar to the service multiplier method [14–16]. Instead of using the number of clients receiving a specific service, a count is created by distributing a memorable object directly to TGW and TGM. Two weeks before the community-based survey, outreach workers distributed a distinct key chain to as many TGW and TGM as they encountered at venues across the nine study districts. In the community-based survey, respondents were asked: “Did you receive one of these key chains [show gift key chain]?” The population size is calculated as the number of key chains distributed ÷ the proportion of survey respondents who report receiving the key chain. The 95% CI is calculated using the general capture-recapture method [15].

### Bayesian synthesis of multiple estimates

A Bayesian process called the Anchored Multiplier developed by Wesson *et al* [21,22] was used to fit the results of the four different methods (i.e., key informant mapping, wisdom-of-the-crowd, service multiplier, and unique object multiplier) into a single best estimate and credible range. The process requires a prior estimate and anticipated distribution to build upon. In the present study, the prior estimate was the full range of all four point estimates, and the distribution was assumed to be uniform.

### Ethical considerations

The study was reviewed and approved by the Research Ethics Board of Health of Bhutan (Ref. No. REBH/Approval/2019/051). The data collectors explained the purpose of the study and procedures. Respondents were informed that their participation was voluntary and had the right to decline to answer any questions or withdraw from the study at any time for any reason. Respondents were also informed that their withdrawal from participation would not impact their access to health care services. Procedures to maintain confidentiality (i.e., anonymity) were explained. Verbal informed consent was obtained before the start of the survey.

## Results

The surveys recruited 34 TGW and 124 TGM (Table 1). The majority (91%) of TGW identified as “transwomen”, with two (6%) identifying as “female” and one (3%) as “other” gender. All TGM (100%) identified as “transmen”. Most TGW (97%) and TGM (99%) reported their sexual identity as “straight”.

**Table 1:** Gender and sexual identities of transgender women and men, Bhutan, 2020.

Characteristic, risk	Transgender women (N = 34)	Transgender men (N = 124)
	n (%)	n (%)
**Sex assigned at birth:**		
Male	34 (100)	0 (0)
Female	0 (0)	124 (100)
Intersex	0 (0)	0 (0)
Don’t know	0 (0)	0 (0)
Other	0 (0)	0 (0)
**Current gender identity:**		
Male	0 (0)	0 (0)
Female	2 (6)	0 (0)
Transwoman	31 (91)	0 (0)
Transman	0 (0)	124 (100)
Other	1 (3)	0 (0)
**Sexual identity:**		
Straight	33 (97)	123 (99)
Gay	0 (0)	0 (0)
Bisexual	1 (3)	0 (0)
Lesbian	0 (0)	0 (0)
Other	0 (0)	1 (1)
**Mean age in years (SD)**	26 (7)	25 (4)

Note: Categories may not add up to the total due to missing data, declined to answer, or don’t know; percentages are among those who responded.

Table 2 presents population size estimates for TGW and TGM by the four different methods and the Bayesian synthesis of the results of these methods. Key informant mapping estimated 113 TGW (range 88–138) and 102 TGM (range 81–120) as the total count of transgender persons affiliated with public spaces in the nine study districts. For the wisdom-of-the-crowd question, the mean response of TGW surveyed was that they numbered 79 (95% CI 49–109) in Bhutan. The mean response of TGM was that they numbered 269 (95% CI 206–332) in Bhutan. The service multiplier (i.e., membership in Pride Bhutan) produced an estimate of 40 (95% CI 29–52) TGW and 87 (95% CI 66–109) TGM in Bhutan. The unique object multiplier produced an estimate of 29 (95% CI 19–40) TGW and 123 (95% CI 102–145) TGM in the nine study districts. The Bayesian synthesis of these four estimates, using the full range of estimates with a uniform distribution as priors, produced 84 TGW (credible interval 61–110) and 166 TGM (credible interval 124–211).

**Table 2:** Population size estimates of transgender women and transgender men in Bhutan, 2020.

Method of size estimation	Estimated number of transgender women (acceptable limits)	Estimated number of transgender men (acceptable limits)
Key informant mapping	113 (88–138)^a^	102 (81–120)^a^
Wisdom-of-the-crowd	79 (49–109)^b^	269 (206–332)^b^
Service multiplier	40 (29–52)^b^	87 (66–109)^b^
Unique object multiplier	29 (19–40)^b^	123 (102–145)^b^
Bayesian synthesis of the above results	84 (61–110)^c^	166 (124–211)^c^

Note

^a^Range

^b^95% confidence interval

^c^credible interval.

Measures of the district of birth and current residence indicated high mobility and rural to urban migration. For example, 8.8% of TGW participants in our survey were born in Thimphu while 50.0% were residents of Thimphu at the time of the survey. Similarly, 9.7% of TWM participants were born in Thimphu while 58.9% resided in Thimphu. Moreover, the 34 TGW respondents were born in 16 of the country’s 20 districts. The 124 TGM respondents were born in 18 of the 20 national districts.

## Discussion

Our study estimates that 84 TGW are living in Bhutan, with a plausible range of 61 to 110. Comparable estimates for similar settings are rare [26]. A recent estimate from Sri Lanka places the population rate of TGW at 0.04% of adult men [14]. Using our estimate, the percent of the population who are TGW is 0.03% of adult men in Bhutan (or equally 0.03% of adult women). Our estimate is also in agreement with the UNAIDS recommendation that estimates of TGW are likely to be 0.02% (25^th^ percentile 0.02%, 75^th^ percentile 0.06%) of adult men in Asian countries [13,14].

Our estimate of 166 TGM indicates that the population comprises 0.06% of adult men in Bhutan. To our knowledge, our study is the first to measure the population size of TGM in the region. Our estimated number of TGM in Bhutan is notably higher than TGW, at a ratio of two-to-one. A population-based estimate in the United States places the ratio closer to one-to-one [27,28], with slightly more TGW than TGM (i.e., TGW are 53.8% of transgender persons). The interpretation that there are more TGM than TGW in Bhutan is bolstered by the fact that we were able to recruit far more TGM in the community-based surveys and that three of the four size estimation methods found substantially more TGM than TGW. The one exception is key informant mapping, which is based on visibility in public spaces, estimating nearly equal numbers of TGM and TGW. A hypothesis on why there may be more TGM offered by key informants is that men enjoy a higher status than women in society. Confirmation of the finding of more TGM than TGW in Bhutan and reasons why need to be explored in future research.

We acknowledge multiple limitations inherent in our study’s methods and resulting from challenges in the field. In the absence of a true census of transgender persons or a gold standard method against which to compare, we rely on the application and synthesis of multiple methods to arrive at the most robust estimates that are feasible. Nonetheless, each method has potential biases. A limitation of the key informant mapping and wisdom-of-the-crowd methods is that they entail subjectivity that is difficult to validate. Key informant mapping is dependent upon the perceptions of stakeholders about the visible part of the population, while the wisdom-of-the-crowd method is based on perceptions of community members themselves. These methods also depend upon informants to de-duplicate their estimation; for example, accounting for venues that see the same clientele. Non-independence of the service count and unique object distribution from participation in the survey threaten the validity of the multiplier methods [15]. Positive dependence results when persons receiving the service or the object have an increased likelihood of also participating in the survey. The net effect of this bias is to reduce the population size estimate. Notably, the multiplier methods generated the lowest estimates for TGW. Adjustment can be done through multiple sample capture-recapture methods that model dependence when three or more overlaps between sources are available [15,16]. In our data, unfortunately, there were too few overlaps between the survey, the services, and receiving the unique object to model and correct for dependence. Other potential biases of the multiplier methods are duplicate counts and misidentification of transgender persons for the multiplier methods. To minimize this bias, the service count used unique registrants and the object distribution occurred in as short a period as possible and relied on peer outreach workers to recognize previous participants. Misalignment of the geographic area of the study with the whole of the country also affects our overall population size estimates. Namely, the key informant mapping and the unique object multiplier method were applied to the nine study districts whereas the wisdom-of-the-crowd question and the service count were applied to the whole country. We believe the difference in numbers between the nine study districts and the whole of Bhutan is likely small as these are the most populous and many adult transgender persons reported migrating to larger cities in our survey.

We posit that our results refer to the more visible and reachable part of the transgender populations of Bhutan, leaving out those who are less visible and residing in rural areas. Stigma may also create biases across all our methods to the extent that many transgender persons may not identify themselves as such in public or online spaces. We feel the net effects of the potential biases are likely to result in an overall underestimation of the total population of TGW and TGM in Bhutan. This assessment of underestimation is bolstered by the fact that three of the four methods estimated fewer TGW and TGM in the country than we were able to recruit in the surveys.

In summary, we triangulated multiple methods to estimate the number of transgender persons in Bhutan for the first time. Estimates fall within rates expected for transgender persons in the region of South and East Asia. Nonetheless, we believe our estimates are low considering the potential biases. Estimating the numbers of small, stigmatized, and hard-to-reach populations is difficult throughout the world, but the attempt is necessary to set appropriate targets for programs to achieve the goals of ending the HIV epidemic by 2030 [29]. Our data are also important from an advocacy perspective in documenting the presence and minimum numbers of TGW and TGM in the current society of Bhutan. Our study documented that TGW and TGM are part of Bhutanese society, and are born in all regions of the country, with TGW constituting three per 10,000 adult women and TGM six per 10,000 adult men. Estimates can help advocate for resources and programs to address the health and well-being of these marginalized communities.
